# Signaling Transduction Network Mediated by Tumor Suppressor/Susceptibility Genes in NPC

**DOI:** 10.2174/138920209788488481

**Published:** 2009-06

**Authors:** Minghua Wu, Xiayu Li, Xiaoling Li, Guiyuan Li

**Affiliations:** Cancer Research Institute, Central South University, Hunan, the People’s Republic of China

## Abstract

Nasopharyngeal carcinoma (NPC) is a polygenetic disease. SPLUNC1, UBAP1, BRD7, NAG7, NOR1, NGX6 and LTF genes were found to be tumor suppressor/susceptibility genes in different stages of NPC. SPLUNC1, an early warning molecular diagnosis marker, inhibits the bacteria clone formation, and is an innated immune molecule. SPLUNC1 can negatively regulate the ERK/MAPK signaling transduction pathway to inhibit NPC cell proliferation and induce apoptosis. BRD7, a transcript regulation factor, interacts with BRD2, and promotes apoptosis induced by BRD2. Its promoter is regulated by c-Myc and SP1. BRD7 inhibits NPC cell cycle progression, preventing passage through G0/G1 by suppressing ras/MEK/ERK, Rb/E2F and Wnt signaling pathways. Abnormal activation of BRD7 is crucial to cell cycle turbulence in NPC. NGX6, a metastasis-associated protein, can negative-regulate the EGF/Ras/MAPK signaling transduction pathway, and interacts with ezrin protein to inhibit NPC cell invasion and metastasis. LTF, also a metastasis-associated protein, can negatively regulate MAPK signal transduction pathways, such as JNK2 and ERK, to inhibit NPC cell proliferation and growth. Taken together, it was found that these tumor suppressor/susceptibility genes can regulate key molecules involved in cell signal pathways such as ras/MEK/ERK, Rb/E2F and EGFR ras/MEK/MAPK, and can regulate the expression of some adhesion molecules such as ezrin, nm23 and α-catenin. According to functional genomics and signaling transduction pathways, we have described a signaling cross-talk network between the tumor suppressor/susceptibility genes involved in NPC. These tumor suppressor/susceptibility genes may be potential treatment targets for NPC in the future.

## INTRODUCTION

**Fig. (1):**
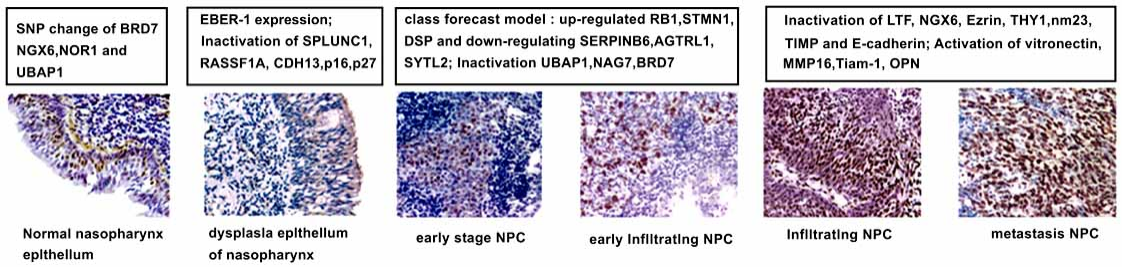
Model of molecular markers involved in the multi-step process of NPC [5].

Nasopharyngeal carcinoma (NPC) is a multifactorial disease that presents a challenge to clinicians and biologists in various fields including epidemiology, genetics, virology and immunology [1]. NPC has a striking geographical distribution and Southern China is a major nasopharyngeal carcinoma-endemic region. Chinese emigrants continue to have a high incidence of the disease, but the rate of NPC among ethnic Chinese born in North America is considerably lower than those born in China [2]. This epidemiologic evidence implies that both environmental factors and genetic susceptibility play roles in the development of NPC. The possible existence of several susceptibility genes (including two of them on the 4q [3] and 3p [4] chromosomes) pointed to the importance of epidemiology and genetics in NPC. Cellular gene alterations also contribute to NPC development, especially inactivation of tumor suppressor genes, such as SPLUNC1, UBAP1, BRD7, NOR1, NGX6 and LTF, which are involved in different stages of NPC initiation and progression (Fig. **1**) [5]. 6 SNP of BRD7 (C450T and A737C), NGX6 (rs879284), UBAP1 (rs1049557) and NOR1 (ss2220003 and ss3211583) have been demonstrated to be important genetic susceptibility risk factors for NPC [6-9]. Xiao *et al.* found that the expression of UBAP1 decreases during NPC development and progression from normal epithelium of nasopharynx to hyperplastic epithelium of nasopharynx, then, to atypical hyperplastic epithelium of the nasopharynx and finally, to nasopharygeal carcinoma [5].

Although combination radiotherapy/chemotherapy is sensitive for stage I and II NPC and improves survival rates, unfortunately the majority of NPC is diagnosed in advanced stages because of nonspecific presenting symptoms (cervical nodalenlargement, headache, nasal and aural dysfunction), delay in seeking treatment after the onset of symptoms, and the difficulty of a thorough nasopharyngeal exam [10]. In light of this, more precise molecular diagnosis and targeted treatments of NPC need to be developed. In this review, the function of tumor suppressor/susceptibility genes in different stages of NPC and signal transduction mediated by tumor suppressor/susceptibility genes will be discussed. Specifically, we will provide evidence for molecular diagnosis, prognosis and targeted treatments in NPC.

## SPLUNC1, AN EARLY WARNING MOLECULAR DIAGNOSIS MARKER, IS INVOLVED IN ERK/MAPK AND NF-κB SIGNALING PATHWAYS, WHICH INDUCE CELL APOPTOSIS IN NPC

SPLUNC1 (short palate, lung, and nasal epithelium clone 1) is isolated from human nasopharyngeal epithelia by suppression subtractive hybridization (SSH) and cDNA microarray analysis [11]. Its expression is downregulated in 70% of nasopharyngeal carcinoma (NPC) biopsies but upregulated in 25% of lung cancer biopsies. SPLUNC1 is a gene transcript of the PLUNC gene family and also is a member of the BPI (bactericidal permeability increasing protein)/LBP (lipopolysaccharide-binding protein) family with putative bactericidal/bacteriostatic functions. All BPI and LBP, including SPLUNC1 are bactericidal proteins and have the function of neutralizing endotoxin [12-14]. SPLUNC1 is downregulated in slight atypical hyperplasia of nasopharynx endepidermis, and its expression gradually decreases with progression of malignant nasopharynx endepidermis from slight atypical hyperplasia to nasopharyngeal carcinoma [15]. It is found to scavenge bacterial endotoxin [16], EB virus [16] and it also can combine with nanobacteria [17] and ameliorate its deleterious effects. Furthermore, it is involved in the earliest stage of NPC carcinogensis, where it plays a role in innate immunity. Thus, SPLUNC1 is an early warning molecular diagnosis marker and is used to carry out early diagnosis and crowd risk forecasting for NPC.

The MAPK (mitogen-activated protein kinase) cascade relays the presence of extracellular stimuli such as growth hormones to the nucleus and controls the expression of hundreds of genes. MAPKs control major cell fate decisions such as proliferation, differentiation and apoptosis, mainly by inducing alterations in gene expression [18]. The MAPK cascade includes four main signaling pathways, ERK, JNK, P38 and ERK5. SPLUNC1 enhances apoptosis of NPC HNE1 cells and EBV-transformed human B-lymphocytes [16]. SPLUNC1 evidently inhibits the expression of phosphorylated ERK (p-Tyr-204) and its BPI domain contributes to this inhibitory effect. SPLUNC1 inhibits the expression JNK2, not JNK1, and has no effect on total P38 protien or phosphorylated P38 (p-Tyr-182). At the same time, SPLUNC1 can promote expression of NF-κB (Nuclear factor NF-κB) and I-κα protein, which is an inhibitory effect dependent upon its BPI domain, but the inhibitory effect of phosphorylated I-κα(p-Ser-32) has nothing to do with its BPI domain. Therefore, SPLUNC1 can regulate ERK/MAPK and NF- kB signaling pathways to induce cell apoptosis in NPC [15].

## DOWNREGULATED EXPRESSION OF RASSF1A IS INVOLVED IN NF-κB SIGNALING PATHWAY

Ras-association domain family of proteins (RASSF) are characterized by the Ras-association (RA) domain at the C-terminal. RASSF1A is a target tumor suppressor gene on 3p21.3 in NPC. The aberrant hypermethylation of RASSF1A and high EBV load might be important events in NPC pathogenesis and they may be useful molecular diagnostic markers for this cancer [19-21]. Downregulation of RASSF1A expression is dependent on the activation of intracellular signaling of NF-κB involving the C-terminal activating regions (CTARs) of LMP1 [22].

## BRD7, A NUCLEAR TRANSCRIPTION FACTOR, REGULATES CELL CYCLE PROGRESSION BY MEK/ERK/MAPK, Rb/E2F AND Wnt/β-CATENIN PATHWAYS

Cell cycle progression is a strictly controlled process and it needs precise interplay between many molecules. The regulatory pathway controlling G1–S is the Rb/E2F pathway: in the presence of extracellular growth-stimulatory signals, cyclinD1 and its kinase partner cdk4 form an activated complex and phosphorylate Rb and Rb family members, thus inactivating Rb and allowing E2F/DP transcription factors to exert their transactivation activity. This in turn leads to transcription of a number of genes essential for DNA replication and entry into S phase, thus the cell enters into S phase and completes the irreversible cell cycle progression [23]. BRD7 is a nuclear transcription regulation factor containing a bromodomain that is found in many chromatin-associated proteins and in nearly all known nuclear histone acetyltransferases (HATs) and has been found to play an important role in chromatin remodeling and transcriptional activation [24]. The transcriptional regulation of BRD7 is achieved by binding to acetylated histone H3 at Lys14. The bromodomain is essential for this role. Chromatin remodeling, not chromatin modification, is the major mechanism of BRD7 mediated gene transcription [25]. The downexpression of the BRD7 gene has been shown to be critical to the pathogenesis of NPC [26]. Promoter methylation inhibits BRD7 expression in NPC [27] and its promoter is regulated by c-Myc and Sp1 [28]. BRD7 interacts with BRD2 and stimulates apoptosis induced by BRD2. BRD7 protein inhibited cell growth and cell cycle progression from G1 to S phase by transcriptionally regulating some cell cycle associated genes such as Rb and E2F3 in NPC cells [29]. Zhou *et al.* [29] and Peng *et al.* [30] found that potential targets of BRD7 were mainly involved in MEK/ERK/MAPK, Rb/E2F and Wnt/β**-**catenin signaling pathway.

Extra-cellular signals can be transmitted into the nucleus cell cycle machinery through diverse signaling pathways, among which MEK/ERK/MAPK is one intensively studied pathways [31]. The over-expression of BRD7 in NPC cells results in the downregulation of the c-jun, p-MEK and p-ERK1/2 expression and Ap-1 promoter inactivity, therefore, BRD7 plays a negative role in the MEK/ERK/MAPK pathway [29]. E2Fs and DPs protein are both transcription factor families that can form heterodimers that play central roles in the expression of genes essential for S phase entry [23]. The expression of DP2 and E2F3 were both decreased by the induction of BRD7, peaking 4.0- and 6.0-fold, respectively [29] and the transcription targets of E2F/DP, such us replication factor A (RFA), replication factor C37 and 38 subunit (RFC37 and RFC38), were all downregulated as a result of the decrease of E2F3/DP2. RFA and RFC are important components for DNA replication initiation by contributing to origin unwinding, primering and firing during DNA replication [32]. BRD7 interacts with Ceap-16 (centrosome associated protein-16, also termed BLOS2) by C-terminus of BRD7 and the central region of Ceap-16. Through this binding, Ceap-16 can translocate from cytoplasm to the nucleus where it selectively inhibits the transcriptional suppression activity of BRD7 towards certain target genes including E2F3 and cyclin A [33]. In addition, Kim *et al.* [34] reported that BP75, the most homologous gene of BRD7 (bromodomain containing protein 7), was found to bind dishevelled-1 and enhance Wnt signaling by inactivating GSK-3β (glycogen synthase kinase-3 beta). In NPC cells, the induction of BRD7 increased the expression of α-catenin which “hold” β-catenin in the complex and inhibit β-catenin accumulation in the nucleus to induce the downregulation of cyclinD1, E2F3 [30]. As a nuclear transcription regulation factor, the nuclear localization of BRD7 is critical for the expression of cell cycle related molecules and cell biological function. NLS (nuclear localization signal) is an essential motif affecting BRD7 nuclear distribution, and NLS-deleted BRD7 shifts the nuclear localization mostly to the cytoplasm, and failed or reduced to negatively regulate the expression of cell cycle related molecules, cyclin D1 and E2F3, and cell cycle progression from G1 to S phase [35].

## NGX6, A METASTASIS-ASSOCIATED PROTEIN, CAN NEGATIVE-REGULATED EGF/Ras/MAPK SIGNALING PATHWAY AND INTERACT WITH EZRIN PROTEIN TO INHIBIT INVASION AND METASTASIS OF NPC CELLS

Cancer metastasis is a very complicated biological process involving many sequential steps. Cell-cell and cell-extracellular matrix (fibronectin, laminin, collagen, etc.) interactions are involved in the metastatic process [36]. NGX6 (nasopharyngeal carcinoma (NPC)-associated gene 6) is a metastasis-associated gene located on human chromosome 9p21–22. Loss of NGX6 expression is associated with lymph node or distance metastasis in colorectal carcinomas [37] and NPC [38]. The NGX6 protein includes two transmembrane (TM) regions. The extracellular region contains one epidermal growth factor (EGF)-like domain and three potential N-glycosylation sites, and the short cytoplasm (CYTO) contains a tyrosine residue that is a potential tyrosine kinasephosphorylation site. The EGF-like domain is a sequence of 40 aa residues long, which has a significant homology to EGF. The EGF-like domain, which contains a unanimous sequence of CX7CX 2–3GXCX10–13 CXCX3YXGXRCX 5–n, is involved in the interaction between receptors and ligands in cell adhesion and signal transduction [39]. Molecules containing EGF-like domains are mostly involved in cell adhesion, matrix formation, injury, recovery and chemotaxis [39]. N-Glycosylation sites are signatures of some cell adhesion molecules [38]. NGX6 modulates the adhesion and invasion process *via *both the EGF-like domain and the CYTO region [40], and could delay cell cycle G0-G1 progression and thus inhibit cell proliferation by negatively regulating the EGFR Ras/Mek/MAPK pathway in NPC cells. As an EGF-like domain gene [41], NGX6 influences the expression of some important cell adhesion-related molecules. In NPC cells, the expression of RhoA and vitronectin are both decreased following the induction of NGX6, and the induction of NGX6 up-regulates the expression of KRT1, integrin a2, integrin b7, PSCD2L, CD9, ezrin, nm23-H1, VE-cadherin and catenin a2. RhoA is implicated in the invasion of human microvascular endothelial cells (HMEC-1). Ectopic expression of active-RhoA GTPase induces the expression of the MMP-9 metalloproteinase [38]. Vitronectin can promote cell adhesion and spreading. In normal tissue, vitronectin has a homogeneous periductal occurrence, with local accumulations much lower than in carcinoma tissues [38]. KRT1 is a member of the keratin gene family associated with differentiation [38]. Integrins are a family of transmembrane glycoproteins that participate in a wide range of cellular events including cell adhesion, proliferation, apoptosis, differentiation and cell-surface-mediated signaling [38]. PSCD2L (Cytohesin-1) can specifically interact with CD18 (integrin b2) and can promote cell adhesion to ICAM1 [38]. CD9 is a cell-surface glycoprotein, that can modulate cell adhesion and migration and that can also trigger platelet activation and aggregation [38]. Nm23-H1 is a tumor metastasis inhibitor in many tumors [38]. VE-cadherin is a calcium-dependent cell--cell adhesion glycoprotein [38]. The up or down-regulation of those adhesion molecules reflects a possible role of NGX6 in tumor invasion and metastasis.

Ezrin has been suggested to be a mediator of cell motility, is essential for the maintenance of cell-cell adhesion, and is inhibitory towards cell matrix adhesion in human colonic epithelial cells. Ezrin, a linker between membrane protein and cytoskeleton, plays an important role in cell morphology, cytoskeleton reorganization, adhesion, invasion and metastasis. NGX6 can interact with ezrin *via *its cytoplasmic region [38, 42]. NGX6 and ezrin expression is negatively correlation in tissue from NPC biopsies, and the positive ratio of ezrin expression may be associated with the clinicopathological phase [42]. Ezrin is an important promoting factor in the development and metastasis of NPC. The positive expression ratio of ezrin in NPC patients is greater than that in non-NPC patients, and the positive ratio of ezrin in NPC patients with lymph nodes metastasis was much greater than that in NPC patients without metastasis. Ezrin can also bind to cell adhesion molecules such as CD44, CD43, CD46, ICAM-1, ICAM-2 and ICAM-3, and be coprecipitated with E-cadhein and β-catenin, all of which are implicated in cell migration and metastasis [43]. The interaction of NGX6 with ezrin suggests that NGX6 participates in cell migration and metastasis modulation. NGX6 plays an inhibitory role in the migration and invasion of NPC cells by interacting with ezrin and down-regulating the expression of ezrin and ezrin-related signaling molecules.

## NAG7, AN ESTROGEN RECEPTOR REPRESSOR, STIMULATES THE INVASIVE POTENTIAL OF HUMAN NPC CELLS BY REGULATING OF ERA EXPRESSION AND THE H-ras/p-c-RAF AND JNK/AP-1/MMP1 SIGNALING PATHWAYS

NAG7 (NPC-associated gene-7) located on 3p25.3, also known as ERR-10 (estrogen receptor repressor-10), is down-regulated in many NPC biopsy samples and in the NPC cell line HNE1 [44]. As an estrogen receptor repressor, NAG7 can interact with the estrogen receptor α (ERα), and reduce 17β-estradiol (E2) induced activation of ERα transcriptional activity in transient transfection assays of mammalian cells [45]. ERα is a nuclear transcription factor that regulates gene expression by binding to specific estrogen-responsive elements (ERE) on target gene promoters. ERα promotes breast cancer cell growth, but paradoxically, it also inhibits cancer cell invasion *in vitro* and metastasis *in vivo *[46-49]. ERα plays opposing roles in promoting cancer cell growth and inhibiting its invasion and metastasis. NAG7 is a negative regulator of ER, and has a double effect on proliferation and invasion of NPC cell lines. In NPC HNE1 cells, overexpression of NAG7 inhibits the proliferation by arresting cell cycle progression from G1 to S phase and inducing apoptosis [50-52]; but it increases the adhesion, motility and invasion of cells, both *in vitro* and *in vivo*, by down-regulating ERα expression in a E2-independent manner. NAG7 suppresses ER expression and stimulates cell invasion *via *the H-ras/p-c-Raf and JNK/AP-1/MMP1 pathways [53]. The Ras proto-oncogene, a small GTP/GDP-binding protein, is a central component of mitogenic signaling and is a critical player in cancer progression. It affects the progression of human malignancies by increasing proliferation, enhancing invasion and altering cytoskeletal organization [54]. Activation of a linear pathway Ras–Raf–MAPK kinase (MEK) results in phosphorylation and activation of MAPK [55]. NAG7 overexpression in HNE1 cells increases the expression of H-Ras, which leads to the activation of p-c-Raf, while the expression of K-Ras, N-Ras and total c-Raf is unchanged [53]. *NAG7* gene influences the H-ras/p-c-Raf pathway, leading to MAPK signal activation. MAPKs consist of ERK, c-Jun NH2-terminal kinase (JNK), and p38 cascades. JNK activity is essential for cancer proliferation, transformation, invasion and metastasis. Moreover, a positive correlation exists between JNK and lymph node metastases in breast cancer patients [56-58]. It has been reported that JNK may induce changes in the expression of c-Jun in hepatocellular carcinoma cells, resulting in increased expression and enzymatic activity of MMP1 [59]. Degradation of collagen is one of the most critical steps in the invasive and metastatic processes of cancer progression, and up-regulation of MMP-1 is associated with increased cellular invasion and poor prognosis in patients with colorectal, esophageal and advanced gastric cancers [60, 61]. MMP-1 may also promote cell adhesion and motility by influencing cytoskeletal arrangements through its association with different cell adhesion molecules such as CD44 and integrins [61]. NAG7 significantly increases the expression of JNK2 and c-Jun and down-regulated c-Fos, while leaving ERK and p38 unaffected. It also causes an increase in the transcriptional activation of AP-1, which up-regulates the production of MMP-1 [53]. E2 has been reported to up-regulate MAPK phosphatases in breast cancer cells [62], but NAG7 stimulates the JNK2/AP-1/MMP1 pathway in an E2-independent manner. This suggests that NPC tumorigenesis does not involve estrogen stimulation [53].

## LTF, A METASTASIS-ASSOCIATED PROTEIN, CAN NEGATIVE-REGULATE THE MAPK SIGNALING TRANSDUCTION PATHWAY

LTF (Lactotransferrin, also referred to as lactoferrin, LF) is a secreted iron-binding glycoprotein that defends against microbial pathogens in innate immunity [63]. Recently, LTF has been found to have antitumor activity thus regulating tumorigenesis [64-66]. LTF is identified from NPC susceptibility locus in chromosome 3p21.31-21.2, which is a site of frequent loss of heterozygosity in NPC [67, 68]. The expression of LTF is downregulated in NPC biopsy samples [68], and is negatively associated with the progression and metastasis of NPC. LTF is expressed at a significantly lower frequency at the T3/T4 stage or in NPCs with local lymph node metastasis compared to that of NPC at early stages or without metastasis [67]. LTF may act as a tumor suppressor, downregulating the progression and metastasis of NPC. Induction of LTF gene expression in NPC or modulation of cyclin D1, p21 and p27 expression, as well as Rb phosphorylation and signaling through the MAPK pathway may inhibit the development and progression of human NPC [67]. LTF treatment inhibits cyclin D1 expression and Rb phosphorylation, and increases levels of p21 and p27 expression. Upregulation of p21 and p27 may downregulate the expression of cyclin D1 and Rb phosphorylation contributing to NPC growth inhibition [67]. LTF treatment for 12 or 24 hr dramatically reduces expression levels of JNK2 and c-Jun in addition to ERK1/2 phosporylation or c-fos expression, respectively. Extending treatment for longer periods resulted in a continued decrease in the levels of p-ERK1/2 and c-fos expression in 5–8F NPC cells *in vitro*. However, LTF treatment does not modulate expression levels of total ERK1/2, STAT3 and p53 and STAT3 phosphorylation [67]. Inhibition of NPC cell proliferation by LTF is partially mediated through modulation of the MAPK signal transduction pathway, but is independent of p53 and STAT3 signaling.

## THE INCREASED ACTIVITY OF p16 AND p27 BY SUPPRESSOR/SUSCEPTIBILITY GENES IS RELATED TO MULTIPLE SIGNALING CASCADES

Both p16 and p27 are cyclin-dependent kinase inhibitory proteins (CKI) that suppress activity and are frequently inactivated in NPC. p16 negatively regulates cyclin D1 activity to suppress CDK4, subsequently to control the G1/S checkpoint; and phosphorylated p27 allows the cdk2/cyclin E complex to remain activated allowing for cell cycle progression [10]. A majority of NPCs exhibit low level expression of p16 and p27 in dysplasia epithelium of nasopharynx. Suppressor/susceptibility genes increase activity of p16 and p27 by suppressing multi-signaling cascades, such as ERK/ MAPK, JNK/c-Jun, Rb/E2F.

**Fig. (2):**
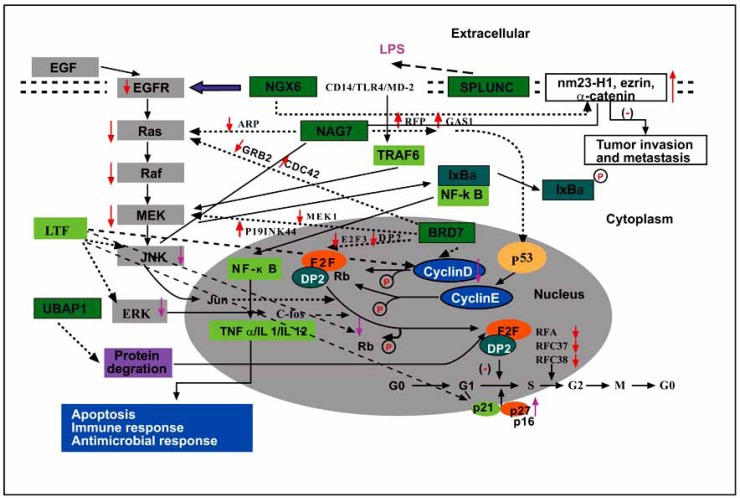
Signaling transduction pathways and cross-talk networks between the tumor suppressor/susceptibility genes involved in NPC

In conclusion, the development and progression of NPC involves in the alteration in expression of numerous oncogenes or tumor suppressor/susceptibility genes and the aberrations of a large variety of signaling pathways. According to functional genomics and signaling transduction pathways, we have described a signaling cross-talk network between the tumor suppressor/susceptibility genes involved in NPC (Fig. **2**). These tumor suppressor/susceptibility genes may be potential treatment targets for NPC in the future.
